# Chicago

**Published:** 1899-08

**Authors:** Jas. B. Clancy

**Affiliations:** Secretary


Secretary.

### CHICAGO VETERINARY SOCIETY.

President Robertson called the regular monthly meeting to order on Thursday evening, May 11th, and directed his remarks toward the important question of legislation and the veterinary bill that was passed at the last session of the Legislature. He is satisfied the act is the best that could be obtained, and regretted the savage attack upon it at the hands of the veterinary press at such a time. The present act, through the State Board of Veterinary Examiners, will bring the veterinarian closer to the legislative and executive officers of the powers that be, thereby enabling them through this closer communion to present the needs of the profession with a more telling effect than has been our good fortune in the past, every effort having been exhausted for the past half-score of years in seeking legislation beneficial to the veterinary profession, and no results until now. The progress that has been made is reason for rejoicing at present, and fills us with hope that the future will be productive of better results than have been attained in the past few years.

Dr. L. A. Merillat explained that there was very little opposition to the measure, there being but three dissenting votes in the Senate and seven in the House.

On a motion presented by Dr. O. R. Dubia it was resolved to extend a vote of thanks to Drs. Robertson and Merillat.

The Board of Censors reported favorably on the application of Dr. Joseph Donovan, and his election followed.

A communication was received from Dr. H. D. Gill, Chairman of the Committee of Arrangements of the A. V. M. A.; it was read and laid over for consideration at the June meeting.

Dr. O. R. Dubia presented a paper which brought out a very interesting discussion.

Adjourned. JAS. B. CLANCY,
Secretary.

### DR. O. R. DUBIA'S PAPER ON SOUNDNESS.

*Sesamoiditis.* This should mean inflammation of the sesamoids, but is applied to other structures in that region, and may be ostitis, ligamentitis, or synovitis—any or all of them. Conformation predisposes to this disease, as shown by certain families with long pasterns and great speed. There is lameness; palpation reveals thickening and tenderness of the fetlock, and on trotting little or no flexion of the fetlock, the knee compensating in part for the loss; it is more common in the hind legs. The soundness of the animal depends somewhat on the part affected, but at best I would call such an animal only "serviceably sound." Hunters and hurdle-horses are commonly affected.

*Windgalls.* This is a distention of the sesamoid sheath which extends two inches above the fetlock, or to the bifurcation of the suspensory ligament. It is due to hypersecretion of synovia and the absence of any rigid structures to prevent distention laterally. This condition cannot be called an unsoundness unless it causes lameness, which it does when distention from accumulation of synovia gives rigidity of the sheath. This is readily determined by forcing the animal to stand on the affected leg, when palpation reveals the amount of rigidity. They are common to all horses, but especially racers. When soft to the touch it is not an unsoundness.

*Anterior Bursal Enlargements.* This condition is seen only on the hind leg, in the bursæ of the extensor pedis tendons in the front of the fetlock. It is generally due to falls, stumbling, or knuckling. When received they cause very acute lameness, swelling, and pain. As the condition, even when chronic, readily yields to treatment, the patient might be called serviceably sound.

*Thickening of the Fibrous Structures.* This subject is almost too indefinite for consideration, including, as it does, all the structures of the region except the osseous, vascular, nervous, and epithelial.

*Knuckling.* Knuckling is an abnormal forward deviation in the direction of the phalanges, but must not be confounded with straight pasterns. It is often symptomatic, or may be due to contraction of the flexors. The diseases and conditions which may cause this trouble are too numerous to mention. It may be idiopathic, as seen in those animals with upright pasterns. This is especially true when the animal is overworked or subjected to faulty shoeing. It is always an unsoundness.

*Interfering from Malformation.* This is a condition in which the moving leg strikes or cuffs its fellow. Is mostly seen in animals of narrow chest or with a faulty conformation of the leg or foot. Weariness, weakness, overwork, or faulty shoeing may be exciting causes. The striking may be slight or only enough to part the hair, or it may be sufficient to cut and bruise the part and cause lameness. When due to malformation it is an unsoundness, but when the exciting causes prevail it should be dealt with judiciously in justice to all concerned.

*Ringbone.* This is an exostosis on the os corona, os suffraginis, or both. It often causes persistent lameness, but the inflammation aborts as ankylosis sets in; movement is limited and permanent stiffness results. They are variously classified according to location as "high" and "low;" and, again, they may be divided into "true" and "false." The latter are often due to rickets, and for use should not be called an unsoundness, but must not be overlooked in selecting a breeding animal. True ringbones may be divided into articular, or those involving the gliding surface, and periarticular, or upon the joint, and is not as serious as the articular form. Ringbones must always be considered an unsoundness, because they impair the usefulness of an animal.

*Sprain of the Inferior Suspensory Ligaments.* These ligaments are commonly known as X, Y, and V ligaments, and are located posteriorly to the pastern. When sprained severely they cause a thickening of the cortex. A sprain of these structures is usually caused by speeding, jumping, or desperate traction. Animals so affected are unfit for fast or heavy work, and are therefore unsound, slight sprains which terminate favorably in a few days excluded.

*Discussion.*

Dr. Hawley: I understood Dr. Dubia to say that a windgall if soft is not an unsoundness. I have seen windgalls as large as a hen's egg, and though the horse showed no lameness, and the windgalls were soft and the horse was capable of performing services all right, still, it being a blemish, I cannot consider the horse sound.

Dr. Dubia: I called such a horse "serviceably sound."

Dr. Hawley: Has this society adopted the expression "serviceably sound?" If so I want to know when the terms "sound" and "serviceably sound" should be used.

Dr. Robertson: The agreement made when we first opened this discussion was that we were to continue the work until all the topics were presented and discussed, and then endeavor to come to some understanding as to when to apply the terms "serviceably sound" and "sound" should be used.

Dr. Merillat: Has Dr. Hawley ever seen a copy of the rules adpoted by the Stockyard Company governing this point?

Dr. Hawley: These rules were formed by Mr. F. J. Berry, but they are of no consequence whatever.

Dr. Merillat: I think it the policy of the Horse Commission Union to call a horse "serviceably sound" that has only such defects as do not materially affect his usefulness or value. This Society should either formulate new rules or revise the old ones, if, as Dr. Hawley says, they are of no value.

Dr. Robertson: Mr. Newgass in his opening address at his sales says there are no perfectly sound horses, and hence the term "serviceably sound."

Dr. Hawley: They have a so-called horse commission union at the yards, who from time to time make rules which all members must adhere to.

These rules are, however, imperfect, and, I believe, in fact, am positive, if this Society would make up a set of rules governing this matter the Horse Commission would accept them. It would be a good plan to do so after this discussion has closed.

Dr. Robertson: I think this is a good suggestion, to revise these rules, and would recommend Dr. Hawley on a committee, as he is familiar with the matter, and, besides, represents both the veterinarians and the horse dealers.

Dr. Hawley: In regard to windgalls, I would like to know if anybody has ever successfully treated a chronic case, *i. e.*, to make the legs as smooth as before their appearance. What is the recognized treatment?

Dr. Merillat: Counter-irritation and aspiration, or both, will, if repeatedly resorted to, cure most any bursal enlargement. Another method, though a dangerous one, is to simply lance them and allow the wound to suppurate. This procedure is always followed by great pain, swelling, and suppuration, from which the patient finally recovers minus the bursal enlargement. I would, however, not recommend such treatment.

Dr. Dyson: I opened up several of them and never had any serious results, and got rid of the windpuffs. I use an antiseptic, and it leaves no scar.

Dr. Robertson: I know of a case that was cured by a trotting-horse trainer by simply bandaging.

Dr. Hawley: I would say that a man who can treat windgalls successfully has "a good thing." I have now a horse in my possession that cannot be sold for a respectable price because of windgalls, as a horse like that is considered second-handed.

Dr. Merillat: Dr. Dyson's treatment for windgalls is indeed remarkable. It is difficult to understand how such a large synovial surface can be exposed to external influences without some of the typical disturbances. The only way to account for it is that his operations were strictly aseptic, and that no invasion was permitted during the healing process.

Dr. Dyson: I have one case at my barn now with two windgalls that I opened, and I will show them to you so that you can judge for yourself.

Dr. Robertson: As to anterior bursal enlargements, the doctor says they are usually found in the hind limb; I have seen very bad cases in the fore limb.

